# Supplementary material for: Single-cell adaptations shape evolutionary transitions to multicellularity in green algae
Source: Nat Ecol Evol. 2023 Apr 20;7(6):889–902. doi: 10.1038/s41559-023-02044-6 (PMC10250200; doi:10.1038/s41559-023-02044-6)
Supplement: Supplementary file 1 — Reporting Summary [file 41559_2023_2044_MOESM1_ESM.pdf]

nature portfolio

Corresponding author(s): Charlie Cornwallis
Last updated by author(s): Mar 1, 2023

Reporting Summary

Nature Portfolio wishes to improve the reproducibility of the work that we publish. This form provides structure for consistency and transparency in reporting. For further information on Nature Portfolio policies, see our Editorial Policies and the Editorial Policy Checklist.

Statistics

For all statistical analyses, confirm that the following items are present in the figure legend, table legend, main text, or Methods section.

n/a	Confirmed	
☐	☒	The exact sample size (*n*) for each experimental group/condition, given as a discrete number and unit of measurement
☐	☒	A statement on whether measurements were taken from distinct samples or whether the same sample was measured repeatedly
☐	☒	The statistical test(s) used AND whether they are one- or two-sided<br>*Only common tests should be described solely by name; describe more complex techniques in the Methods section.*
☐	☒	A description of all covariates tested
☐	☒	A description of any assumptions or corrections, such as tests of normality and adjustment for multiple comparisons
☐	☒	A full description of the statistical parameters including central tendency (e.g. means) or other basic estimates (e.g. regression coefficient) AND variation (e.g. standard deviation) or associated estimates of uncertainty (e.g. confidence intervals)
☐	☒	For null hypothesis testing, the test statistic (e.g. *F*, *t*, *r*) with confidence intervals, effect sizes, degrees of freedom and *P* value noted<br>*Give P values as exact values whenever suitable.*
☐	☒	For Bayesian analysis, information on the choice of priors and Markov chain Monte Carlo settings
☐	☒	For hierarchical and complex designs, identification of the appropriate level for tests and full reporting of outcomes
☐	☒	Estimates of effect sizes (e.g. Cohen's *d*, Pearson's *r*), indicating how they were calculated

Our web collection on statistics for biologists contains articles on many of the points above.

Software and code

Policy information about availability of computer code

Data collection	All data was complied using the open source software R. All versions of the R packages used together with the code and data are available at the open science framework: DOI 10.17605/OSF.IO/B9WPJ.
Data analysis	All data analysis was performed using the open source software R.. All versions of the R packages used together with the code and analysis outputs are available at the open science framework: DOI 10.17605/OSF.IO/B9WPJ.

For manuscripts utilizing custom algorithms or software that are central to the research but not yet described in published literature, software must be made available to editors and reviewers. We strongly encourage code deposition in a community repository (e.g. GitHub). See the Nature Portfolio guidelines for submitting code & software for further information.

Data

Policy information about availability of data

All manuscripts must include a data availability statement. This statement should provide the following information, where applicable:

- Accession codes, unique identifiers, or web links for publicly available datasets
- A description of any restrictions on data availability
- For clinical datasets or third party data, please ensure that the statement adheres to our policy

R code, data and analysis results are available at the open science framework: DOI 10.17605/OSF.IO/B9WPJ. Full citations of references in supplementary tables are given in the method references.

Human research participants

Policy information about studies involving human research participants and Sex and Gender in Research.

Reporting on sex and gender	NA
Population characteristics	NA
Recruitment	NA
Ethics oversight	NA

Note that full information on the approval of the study protocol must also be provided in the manuscript.

Field-specific reporting

Please select the one below that is the best fit for your research. If you are not sure, read the appropriate sections before making your selection.

☐ Life sciences ☐ Behavioural & social sciences ☒ Ecological, evolutionary & environmental sciences

For a reference copy of the document with all sections, see nature.com/documents/nr-reporting-summary-flat.pdf

Ecological, evolutionary & environmental sciences study design

All studies must disclose on these points even when the disclosure is negative.

Study description

We examine the environmental factors explaining the initial evolution of multicellularity in natural systems. We first experimentally examine the environmental factors that induce multicellular group formation across 35 species of unicellular green algae. Second, we test if these environmental factors predict the occurrence of obligate multicellularity across 332 species distributed across 478 lakes over the past 55-years. Specifically, we found that:

• Multicellular groups form in response to multiple environmental drivers, including nitrogen availability, water turbulence and predation.
• Multicellularity did not provide fitness benefits under any condition. Instead, multicellularity was associated with single cells producing extracellular matrix in high nitrogen, turbulent environments with predators that prevented the release of daughter cells.
• The production of extracellular matrix by single cells had a strong effect on fitness indicating that multicellular groups may arise as a by-product of selection on single cell traits.
• Our experimental results corresponded to nationwide patterns across Swedish lake systems. Specifically, extracellular matrix in unicellular species was related to nitrogen availability in lakes and the presence of extracellular matrix was associated with evolutionary transitions to obligate multicellularity.

Research sample

The study experimentally examined 35 species of unicellular chlorophyte algae collected from lakes in Sweden. In addition, long-term monitoring data from 478 Swedish lakes over the past 55-years of 332 species of chlorphyte alage were analysed. All data are provided in Supplementary Tables 1-4.

Sampling strategy

Water samples were obtained close to the shore of 20 southern Swedish lakes (Fig. 1A) using a 15 x 50 cm Apstein net with 10 µm mesh size (Hydro-Bios, Altenholz, Germany) in July and August 2016. The samples were examined for the presence of Chlamydomonas spp. at 100X and 200X in an inverted Nikon Eclipse Ts2 (Tokyo, Japan) microscope. Single swimming cells matching the general description of Chlamydomonas spp. (ca. 10-15 µm in diameter, two flagella and cup-shaped chloroplast) were isolated by micropipetting using disposable glass capillaries (Hirschmann Laborgeräte, Eberstadt, Baden-Württemberg, Germany). Cells were washed in drops of sterile-filtered lake water and placed in 100 µl 1:1 mix of WC medium (Guillard and Lorenzen 1972), modified by 0.002 mg/L Na2SeO3.5H2O (MWC + Se), and filtered lake water in 96-well culture plates (VWR, Radnor, PA, USA). Cultures were maintained at a 12:12 light:dark cycle in 20°C and 85 µmol photons m^-2^ s^-1^, transferred into larger plates as they grew, and finally placed in 25 cm2 non-treated culturing flasks (Thermo Fisher Scientific, Waltham, MA, USA) containing 30 ml MWC+Se medium.

The long-term data used is part of Sweden's national lake monitoring scheme. Details of the sampling strategies can be found at: http://www.slu.se/miljodata-MVM.

Data collection

The experimental data was collected by two post-docs (MSC and ML) and one research assistant (FS).

The long-term data was collected as part of Sweden's national lake monitoring scheme. Details of the sampling strategies can be found at: http://www.slu.se/miljodata-MVM.

Timing and spatial scale

Experimental data: The growth rates of strains and the proportion of cells in multicellular groups were measured at the start (t0), at 48 hours (t2) and at day 14-15 (t14) when the cultures reached approximate stationary phase. The experiments ran from December 2017 to February 2018.

	The long-term data involved sampling lakes across Sweden (n=478) that started 55-years ago. Full details of the number of times and when lakes were sampled is given in the supplementary materials available at the open science framework: DOI 10.17605/OSF.IO/B9WPJ.
Data exclusions	No data were excluded from the analyses.
Reproducibility	All data analysis was performed using the open source software R. All versions of the R packages used together with fully reproducible R project scripts are available at the open science framework: DOI 10.17605/OSF.IO/B9WPJ.
Randomization	Our experimental designed selected strains of algae that had different geographic and phylogenetic history. Within these restrictions we randomly choose strains to study.
Blinding	Experimenters were blind to the treatments when collecting data. In addition, phenotypic measurements were automated using machines (e.g. FlowCam).

Did the study involve field work? ☐ Yes ☒ No

Reporting for specific materials, systems and methods

We require information from authors about some types of materials, experimental systems and methods used in many studies. Here, indicate whether each material, system or method listed is relevant to your study. If you are not sure if a list item applies to your research, read the appropriate section before selecting a response.

Materials & experimental systems

n/a	Involved in the study
☒	☐ Antibodies
☒	☐ Eukaryotic cell lines
☒	☐ Palaeontology and archaeology
☐	☒ Animals and other organisms
☒	☐ Clinical data
☒	☐ Dual use research of concern

Methods

n/a	Involved in the study
☒	☐ ChIP-seq
☒	☐ Flow cytometry
☒	☐ MRI-based neuroimaging

Animals and other research organisms

Policy information about studies involving animals; ARRIVE guidelines recommended for reporting animal research, and Sex and Gender in Research

Laboratory animals	NA. The study is on algae.
Wild animals	NA. The study is on algae.
Reporting on sex	NA.
Field-collected samples	Yes. Chlorophyte algae were collected from natural lakes.
Ethics oversight	NA

Note that full information on the approval of the study protocol must also be provided in the manuscript.
